# 

**Published:** 1902-07-05

**Authors:** 


### NOTICE TO CORRESPONDENTS.

All MSS., Letters, Books for Review, and other matters intended for the Editor should be addressed The Editor, "The Hospital," The Hospital Building, 28 & 29 Southampton Street, Strand, W.C.
The Editor cannot undertake to return rejected MS., even when accompanied by stamped directed envelope.

**Notice to Advertisers and Subscribers.**

All Advertisements, Orders for Copies of The Hospital, and Business Communications should be addressed to **THE MANAGER, (Not to the Editor)**, The Hospital, 28 & 29 Southampton Street, Strand, London, W.C.
The Cost of Subscription, post free, is as follows.—Per week, 2½d.; per quarter, 2s. 8d.; per half-year, 5s. 3d.; per annum, 10s. 6d. Foreign:—Per annum, 15s. 2d., payable, in all cases, in advance.

**NOTICE.—Vol. XXXI. of "The Hospital" (October 1901, to March 1902), in handsome cloth binding is now on sale at the office of this Journal, price, 7s. 6d. Cases for binding the Numbers contained in this Volume 1s. 6d. Index to Vol. XXXI., 2½d., post free.**
**The Monthly Part for July is now ready. Price 6d., by post 8d.**

### SCRAPS AND GLEANINGS.

THE plans of Mr. A. E. Painter, of Wolverhampton, have been accepted for the proposed new hospital for women to be erected in Park Road West.

THE Stockport Amateur Dramatic Society have given £20 towards the funds of the Stockport Infirmary, being the result of the society's performances during the past season.

Two performances of "Caste" were given recently at the Royal Albert Hall Theatre, in aid of "the fund for supplying lamps for the light-cure of lupus at the London Hospital." The performances were under the direction of Miss Elsie Fogerty, and the cast included Mr. Stephen Hawtrey.

THE new Grace Swan Cottage Hospital at Spilsby is an extension of the movement undertaken six years ago to raise a memorial to the late Mrs. Swan. This movement resulted in £1,000 odd being raised, with which amount a District Nursing Association was founded. Up to the present four nurses have been employed by the Association, and a year ago it was decided to extend the movement by building a hospital for use by certain patients and to form a home for the nurses. Lord Willoughby de Eresby very kindly gave the site and the Grace Swan Cottage Hospital, containing two wards, and accommodation for a matron, four nurses, and a servant is the result.

THE accounts submitted at the recent annual general meeting of the National Sanatorium for Consumption, Bournemouth, show that there has been a loss on the year's work of £554, a balance in hand of £122 at the commencement of the year having been turned into a deficit of £432. This deficiency is ascribed (1) to a falling off in donations, which were £275 less than they were in 1900; (2) to the withdrawal by the London Hospital Saturday Fund of its grant of £50; and (3) the receipt of £32 less than last year from patients' payments. The committee of the institution urgently appeal for money to enable them to wipe off this deficiency, and also for contributions to the Special Building Fund, which has been started with a view of raising £1,200 required to carry out some additions and alterations urgently needed.

It is proposed to erect a cottage hospital in Oakhampton, providing eight beds, which shall be primarily regarded as intended for the benefit of persons belonging to families whose income does not exceed 25s. per week throughout the year. The joint committee of the Oakhampton Town Council are the prime movers in the matter. They have in hand a capital sum for building purposes of £100, and it is desired to secure the rest by private donations. The maintenance of such a hospital, it is estimated, will cost £150 per annum, and it is confidently anticipated that the proceeds, amounting to about £76 yearly, of some trust money which the Oakhampton Town Council control, will be devoted to this object.

### THE STUDENT OF MEDICINE.

### APPOINTMENTS.

**J. S. Colquhoun**, L.R.C.P., L.R.C.S.Edin., appointed Medical Officer for the South Side of Gairloch Parish. **J. M. Crocker**, M.R.C.S., L.R.C.P.Lond., appointed Certifying Factory Surgeon for the Bingley District of Yorks. **Ramsay Daly**, M.D., B.S., appointed Clinical Assistant to the Chelsea Hospital for Women. **P. J. Heneghan**, L.R.C.S.I., L.A.H.Dub., appointed Certifying Factory Surgeon for the Foxford District of County Mayo. **Charles Henry Hibbert**, L.R.C.P.., L.R.C.S.E., L.F.P. and S.G., appointed Medical Officer of Health for the Urban District of Compstall (Cheshire). **H. Campbell Highet**, M.D.Glasg., D.P.H.Lond., appointed Physician to H.B.M. Legation, Bangkok, Siam. **Maynard Horne**, M.A., M.B., B.C.Cantab., M.R.C.S., L.R.C.P., appointed Assistant Anæsthetist to the Hospital for Women, Soho Square, W. **Rowland Lee**, M.B., Ch.B.Lond., appointed Assistant Medical Officer to the Cardiff Union Workhouse. **Lawrie Hugh McGavin**, F.R.C.S.Eng., appointed Surgeon to In-patients at the Branch Hospital of the Seamen's Hospital Society, Royal Albert Dock. **C. Jerome Mercier**, M.R.C.S.Eng., L.R.C.P.Lond., appointed Resident Medical Officer to the St. Mary's Hospital for Sick Children, Plaistow. **Patrick Murison**, M.B., C.M.Edin., D.P.H.Camb., appointed Medical Officer of Health for the Corporation of Durban, Natal. **E. C. Nangle**, L.S., B.A., appointed Clinical Assistant to the Chelsea Hospital for Women. **Robert A. St. Leger**, M.B.Edin., appointed Honorary Physician to the New Somerset Hospital, Capetown. **Cecil E. Shaw**, M.D., M.Ch., R.U.I., appointed Honorary Consulting Ophthalmic Surgeon to the County Antrim Infirmary, Lisburn. **Henry F. Skrimshire**, M.R.C.S.Eng., L.R.C.P.Lond., appointed Assistant Resident Medical Officer to the St. Mary's Hospital for Sick Children, Plaistow. **Kenneth Steele**, M.R.C.S., L.R.C.P., appointed Anæsthetist to the Seamen's Hospital Society, with duties at the *Dreadnought* Hospital, Greenwich. **F. B. Thornton**, M.B., B.S.Lond., appointed Medical Officer to the Post Office at Derby, and Medical Referee for the Board of Education at the Derby Centre.

### "The Hospital" Institutional Library.

"Hospitals and Asylums of the World." 4 Vols., with a Portfolio of Plans, complete £12 12s.
"Burdett's Hospitals and Charities, 1902." 5s.
"Cottage Hospitals, General, Fever, and Convalescent." 10s. 6d.
"Hospital Expenditure: The Commissariat." 2s. 6d.
"A Legal Handbook for the Use of Hospital Authorities." 2s. 6d.
"The Cottage Hospital Case Book or Register of Patients." 10s. and 12s. 6d.
"The Furnishing and Appliances of a Cottage Hospital." 6d.
All these are published by the Scientific Press, Ltd., and may be obtained through any bookseller, or direct from the publishers, 28 and 29 Southampton Street, Strand, London, W.C.

